# Persistent *Burkholderia pseudomallei* Bacteremia in A Filipino Immigrant to the United States: A Case Report

**DOI:** 10.3390/tropicalmed4010020

**Published:** 2019-01-28

**Authors:** Sumbul Meraj, Brandy Rodenberg, Stephanie Thannum, Jared Sheley, Jena Foreman

**Affiliations:** 1 HSHS St. Elizabeth’s Hospital, O’Fallon, IL 62269, USA; stephanie.thannum@hshs.org (S.T.); jared.sheley@hshs.org (J.S.); jena.foreman@hshs.org (J.F.); 2 School of Pharmacy, Southern Illinois University Edwardsville, Edwardsville, IL 62025, USA

**Keywords:** *Burkholderia pseudomallei*, melioidosis, ceftazidime, resistance, persistent bacteremia

## Abstract

Melioidosis is rare in the United States and endemic to Southeast Asia and Australia. Treatment includes an initial intensive phase of intravenous ceftazidime or meropenem monotherapy depending on severity. The following report describes a case of persistent bacteremia with ceftazidime failure and prolonged meropenem therapy on a ceftazidime-susceptible strain of *Burkholderia pseudomallei*.

## 1. Introduction

*Burkholderia pseudomallei* is a Gram-negative, aerobic bacillus found naturally in the water and soil of many countries in Southeast Asia, the Pacific Islands, and northern Australia [1]. Transmission of this bacterium occurs through inoculation, ingestion, or inhalation. Historically, in the 19th and 20th centuries, this bacterium had been used as a bioterrorism weapon due to its translocation in water leading to infection during monsoon season in areas where it is endemic [2,3]. Infection with *B. pseudomallei,* commonly known as melioidosis, presents as pneumonia in nearly 50% of patients, but may also cause bone, skin/soft tissue, or central nervous system infections. In many cases, the infection progresses to bacteremia that can rapidly become fatal [1,3]. Risk factors for melioidosis include travel to, or residence in, endemic areas, immunosuppression, smoking, chronic lung disease, diabetes mellitus, chronic liver disease, and renal insufficiency [4]. Melioidosis may present as an acute, chronic, or latent infection, and inappropriate treatment or shortened duration of therapy may lead to relapse or re-infection [4]. Relapse rates vary based on patient-specific risk factors, but have ranged from 5%–10%, and the mortality rates following *B. pseudomallei* infection are as high as 34.8%, despite appropriate ceftazidime or meropenem intensive therapy [5]. Although rare in the United States (US), the number of reported cases to the Centers for Disease Control and Prevention continues to grow, with most patients reporting recent travel outside of the US. Due to this rarity of melioidosis in the US, there are no established US treatment guidelines and available treatment information consists of international studies and guidelines. 

Current treatment recommendations in Australia include an intensive phase of intravenous (IV) antibiotics followed by a prolonged oral eradication phase, which minimizes the risk of relapse [6,7]. These recommendations include a minimum of 10–14 days of IV ceftazidime or meropenem followed by a three- to six-month eradication phase using oral sulfamethoxazole/trimethoprim, doxycycline, or amoxicillin/clavulanate depending on the source of infection and other patient-specific factors [8].

Several studies have established ceftazidime monotherapy as the first-line intensive therapy agent. An open randomized trial compared 120 mg/kg/day ceftazidime to conventional therapy, which consisted of the combination of chloramphenicol, doxycycline, and sulfamethoxazole/trimethoprim, and found that ceftazidime reduced mortality from 74% to 37%, comparatively [9]. Ceftazidime monotherapy has also been compared to ceftazidime plus sulfamethoxazole/trimethoprim and was found to have similar rates of both mortality and recurrences [10]. Observational studies have shown that meropenem therapy may be clinically preferable over ceftazidime due to the concerns of potential resistance through penicillin binding protein 3 gene deletions in *B. pseudomallei*, lack of growth of resistant strains on agar plates, and early relapse of bacteremia associated with ceftazidime [11]. Meropenem is currently preferred over ceftazidime in cases of neuromelioidosis, persistent bacteremia, and critically ill patients [12,13]. Currently there is no strong evidence comparing carbapenems and ceftazidime, but there is a randomized, blinded trial comparing ceftazidime to meropenem underway in Thailand [14].

Persistent bacteremia with *B. pseudomallei* correlates to increased risk of death from melioidosis. Therefore, blood cultures should be performed weekly. Repeating cultures from other sites have shown no benefit for prognostic value [15]. Furthermore, when growing *B. pseudomallei,* it is recommended to utilize Ashdown agar in preference to blood agar when available due to potential organism misidentification as *Pseudomonas* or *Burkholderia* spp., which could delay appropriate treatment [15].

## 2. Patient Case

The patient’s written informed consent and Southern Illinois University Edwardsville IRB approval were obtained for this case report. A supplemental table (Table A1) has been provided in Appendix A, which outlines antibiotic therapy and culture reports throughout the patient’s hospital admission. 

Our patient is an 81-year-old, 53 kg, 160 cm tall female from the Philippines who presented to our emergency department in southwestern Illinois with shortness of breath. Her past medical history is significant for asthma, hypertension, dyslipidemia, cerebral aneurysm, and arthritis. She reported moving to the US in 1979 and had recently returned from a one-month long visit to Quezon City, an urban area in the Philippines, five days prior to admission. Risk factors for melioidosis in this patient included: Travel to an endemic country and asthma (controlled at baseline). The patient had no sick contacts during or after her visit to the Philippines. There were no adverse weather events and the patient had no exposure to rural or agricultural areas during her stay.

The patient expressed having increased shortness of breath while ambulating or performing any exertional activities, along with decreased appetite, left-sided chest pain, weakness, fever, cough, and wheezing. Computed tomography (CT) of the chest demonstrated left upper lobe pneumonia, small bilateral pleural effusions, and bibasilar atelectasis. Chest x-ray showed patchy left basilar opacity suggestive of an infiltrate. Blood cultures and a respiratory culture were obtained in the emergency department prior to initiation of ceftriaxone and azithromycin for empiric treatment of suspected community-acquired pneumonia. On admission, her white blood cell (WBC) count was 12,900 cells/µL, temperature was 38.6 °C, and the patient was admitted with an initial diagnosis of pneumonia with acute respiratory distress. 

On day three of admission, WBC count continued to trend up to 20,900 cells/µL while on ceftriaxone and azithromycin. The blood cultures drawn on day one of admission reported growth of *Burkholderia* species in one out of two bottles, and the respiratory culture resulted with no growth as the final result. Based on a creatinine clearance of 48 mL/min, ceftazidime 2 grams IV was initiated at a decreased dosing frequency of every 12 h, instead of every eight h, on day four of admission. 

While on ceftazidime, the blood cultures drawn on day four reported the same growth of *Burkholderia* species. The patient’s WBC count continued to increase, peaking at 33,300 cells/µL, before trending down to 16,200 cells/µL by day eight of admission (day five of ceftazidime therapy). 

On day nine of admission, the patient’s WBC count increased to 18,600 from 16,200 cells/µL, and CT with contrast of the chest was performed and demonstrated progressive complete consolidation of the superior lingular segment of the left upper lobe and new internal cavitation, which was concerning for necrotizing pneumonia. A two-dimensional (2D) echocardiogram did not demonstrate any vegetation at this time. Given the worsening CT findings coupled with persistent bacteremia, therapy was changed to meropenem 1 g IV every eight h.

On day 13 of admission, the regional laboratory reported the results of the culture drawn on day one of admission. Identification and susceptibility were the following: *B. pseudomallei*, susceptible to chloramphenicol, ceftazidime, meropenem, and sulfamethoxazole/trimethoprim. The Centers for Disease Control and Prevention were notified of the results from the regional public health laboratory. A CT of the abdomen and pelvis was performed and did not demonstrate any fluid collection or abscesses. Later that same day, temperature increased to 37.2 °C. Due to identification and susceptibility testing, and decrease in patient’s creatinine clearance, repeat blood cultures were obtained and the dose of meropenem was increased to 2 grams every 12 h, and sulfamethoxazole/trimethoprim (320 mg trimethoprim component) twice daily was added to the therapy regimen. Incidentally, the blood cultures drawn on day 13 were drawn before starting sulfamethoxazole/trimethoprim and returned no growth after a total of 10 days of IV therapy (five days of ceftazidime and five days of meropenem). 

Meropenem was continued for four days after the first negative blood culture was drawn (on day 13 of admission) for a total of 14 days IV intensive therapy. The patient was discharged home on oral sulfamethoxazole/trimethoprim (320 mg trimethoprim component) twice daily for a planned duration of three months. 

Two weeks after hospital discharge, the patient was readmitted due to hyponatremia, hyperkalemia, and acute kidney injury. The sulfamethoxazole/trimethoprim dose was decreased during that admission. The previous dose was resumed on discharge as the admitting diagnoses resolved. Blood cultures repeated during this hospitalization were negative. 

One month after the initial hospitalization, the patient was seen in clinic and remained free of symptoms of infection. No fevers or decrease in oxygen saturation was documented.

One month into treatment with sulfamethoxazole/trimethoprim, the patient developed acute kidney injury with a serum creatinine of 1.30 mg/dL (baseline of 0.64 mg/dL). Due to this adverse effect, therapy was switched to oral doxycycline 100 mg twice daily to complete the remaining course of maintenance therapy. Completion of the antibiotic regimen was confirmed through follow up with the patient in an outpatient infectious diseases clinic. 

## 3. Discussion

Since this patient was not critically ill on hospital admission and had a pulmonary source of infection, ceftazidime therapy was not initiated until blood cultures returned positive for *Burkholderia* spp. Treatment was broadened to meropenem due to lack of clearance of bacteremia after approximately a week of ceftazidime therapy, in the setting of worsening of pneumonia with cavitation on CT. Sulfamethoxazole/trimethoprim was added in addition to meropenem due to a mild fever and one positive blood culture while on meropenem. We later found out that the blood cultures that were drawn immediately before starting sulfamethoxazole/trimethoprim were negative. Thus, microbiological cure was achieved after prolonged persistence of bacteremia on meropenem monotherapy.

Both ceftazidime and meropenem dosing were adjusted based on the patient’s renal function, though guidelines make no recommendation regarding renal dose adjustment for either of these medications in the treatment of *B. pseudomallei* bacteremia. The recommended dosing interval for both drugs is every eight h. According to drug compendia, the recommended frequency is every 12 h, when considering this patient’s creatinine clearance, due to drug elimination being slowed in renal impairment. With the dose adjustment, the patient did receive lower than studied doses of ceftazidime and meropenem [9,16].

The treatment of persistent bacteremia caused by *B. pseudomallei* after failed ceftazidime therapy is not well-studied or mentioned in the international treatment recommendations. While meropenem and ceftazidime have similar mortality rates according to observational studies, meropenem is typically reserved for septic shock and those patients deemed critically ill [12]. One study demonstrated emerging resistance to ceftazidime therapy not evident on susceptibility reports due to the poor growth of the bacteria on typical laboratory culture medium (ex. agar plates) [11]. Our facility’s microbiology laboratory used blood agar and chocolate agar as culture media for this isolate, which typically yields identification of *Burkholderia* spp. or *Pseudomonas* spp. After the specimen was identified as *Burkholderia* spp., by our laboratory, the specimens were sent to a regional public health lab for identification and susceptibility testing. The culture media used by that laboratory was not disclosed to the authors, though Ashdown agar is recommended for culture media. [14] The susceptibility report that we were sent by a regional public health laboratory confirmed susceptibility to ceftazidime; however, the patient remained bacteremic after six days of ceftazidime, prompting the change to meropenem. Given the delay in identification of *B. pseudomallei,* and the lack of proper culture media for testing, meropenem therapy could have been initiated more expeditiously had the resources been in place to identify this organism earlier in the patient’s hospital stay. 

The risk of mortality from infection with *B. pseudomallei* is increased with persistent bacteremia; therefore, it is prudent to complete blood cultures once weekly. [15] In our case, blood cultures were drawn far more frequently than weekly, as the authors were not immediately aware that the pathogen was *B. pseudomallei*. Had identification occurred earlier in the patient’s stay, the ordering of excessive blood cultures could have been avoided, which would have also decreased risk of exposure to laboratory personnel.

Based on our treatment, we are not able to determine whether or not sulfamethoxazole/trimethoprim should be used in combination with meropenem after ceftazidime failure. However, this case does support the use of meropenem monotherapy for the microbiological cure of persistent *B. pseudomallei* bacteremia in cases of clinical ceftazidime failure, despite ceftazidime susceptibility being shown *in vitro*. Further studies will be necessary to determine efficacy of meropenem vs. ceftazidime monotherapy in achieving microbiological cure in persistent bacteremia with *B. pseudomallei*. Furthermore, given the patient’s travel history and the rarity of melioidosis in the United States, it would have been beneficial to have had appropriate identification and susceptibility testing earlier in the patient’s hospitalization so that therapy could have been optimized sooner.

## 4. Conclusions

This is the first case report published on persistent bacteremia with *B. pseudomallei* in the United States. Despite *in vitro* testing confirming susceptibility for ceftazidime against *B. pseudomallei*, we report a case where a patient had persistent bacteremia with ceftazidime monotherapy, with persistent positive blood cultures on initial meropenem therapy. Prospective clinical trials are needed to determine the efficacy of ceftazidime compared to meropenem for microbiological cure.

## Appendix A

**Table A1:** Antibiotic therapy and culture results throughout admission.

Day of Admission ^1^	Culture Results	Antibiotic Therapy
Day 1 (blood culture #1 collected)	--	--
Day 2	--	Ceftriaxone/Azithromycin
Day 3	#1 *B. pseudomallei* (1 out of 2) ^2^	Ceftriaxone/Azithromycin
Day 4 (blood culture #2 collected)	--	Ceftazidime 2 grams Q12H
Day 5	--	Ceftazidime 2 grams Q12H
Day 6 (blood culture #3 collected)	#2 *B. pseudomallei* (2 out of 2) ^2^	Ceftazidime 2 grams Q12H
Day 7	--	Ceftazidime 2 grams Q12H
Day 8	#3 *B. pseudomallei* (2 out of 2) ^2^	Ceftazidime 2 grams Q12H
Day 9	--	Ceftazidime 2 grams Q12H/Meropenem 1 gram Q8H
Day 10 (blood culture #4 collected)	--	Meropenem 1 gram Q8H
Day 11	--	Meropenem 1 gram Q8H
Day 12	#4 *B. pseudomallei* (2 out of 2) ^2^	Meropenem 1 gram Q8H
Day 13 (blood culture #5 collected)	-	Meropenem 2 grams Q12H/Sulfamethoxazole/Trimethoprim DS
Day 14	No growth Day 1	Meropenem 2 grams Q12H/Sulfamethoxazole/Trimethoprim DS
Day 15	No growth Day 2	Meropenem 2 grams Q12H Sulfamethoxazole/Trimethoprim DS
Day 16	No growth Day 3	Meropenem 2 grams Q12H/Sulfamethoxazole/Trimethoprim DS
Day 17	No growth Day 4	Meropenem 2 grams Q12H/Sulfamethoxazole/Trimethoprim DS
Day 18	No growth Day 5	Sulfamethoxazole/Trimethoprim DS

^1^ Intensive IV antibiotic therapy does not include days of ineffective antibiotic treatment for *B. pseudomallei* (days 1–3). DS—double strength; 800 mg sulfamethoxazole/160 mg trimethoprim); Q—every, H—hours (Q12H—every 12 h). ^2^ Identification and susceptibility testing for these cultures did not result until day 13 of hospital admission.
